# On the Dormant Vitality of the Tobacco Seed

**Published:** 1853-10

**Authors:** W. S. Stoakley

**Affiliations:** Northampton county, Virginia


*On the Dormant Vitality of the Tobacco Seed.* By W. S. Stoakley, M. D., of Northampton county, Virginia.

A gentleman in my vicinity informed me that in cultivating his land, last spring, he had occasion to plough it very deep, and in doing so, he came in contact with something beneath the surface, which he found by examination to be a brick wall, the ground-work or cellar of a building. Wondering that he had not discovered this before, (the land having been cultivated for a number of years by him and this having lain there unobserved up to this time,) he came to the conclusion that it might be a bank of excellent manure. He resolved to cut it out. He did so. After digging several feet below the surface, he came to this layer or *stratum* of manure (a mixture of lime and earth) which he immediately carted out into his field. In a short time he observed, where he had placed this manure, a beautiful and thrifty plant, which, after it had attained some size, he found to be a Tobacco plant, the seeds of which must have lain dormant "upwards" of a century. For it has been longer than that since tobacco was raised in this county. *

The farm on which this "subterraneous wall" was found, was *first purchased and paid for*, in *tobacco*, as will be seen from the following note from "Howe's History of Virginia:

" The labor of the colony, which had been for a long time misdirected in the manufacture of ashes, soap, glass, and tar, in which they could by no means compete with Sweden and Russia, and also in planting vines, which require infinite labor and attention, and for which subsequent experiments have indicated the climate to be unfit, was at length directed, by the extended use of *tobacco* in England. almost exclusively to the cultivation of that article. This commodity, always finding a ready price, and affairs being now so regulated that each one could enjoy the fruits of his labor, was cultivated so assiduously as to take the attention of the planters too much from raising corn, so that it became scarce, and supplies had again to be looked for from England, or purchased of the Indians. The fields, gardens, public squares, and even the streets of Jamestown, were planted with tobacco, and this becoming an article of universal desire, it became, to a great extent, the circulating medium of the colony. Not only private debts, but salaries and officer's fees were paid in tobacco; and the statute-book, to this day, rarely mentions the payment of money, that it does not add, as an equivalent, 'or tobacco.' "

Now, you will perceive from this, that it is probable that the seeds of this plant may have lain dormant for more than two hundred years. This wall, with which these seeds were *buried*, was on a portion of the farm that had been cultivated by past generations, and had not been discovered up to this time. Is it not possible that they may have lain buried from the first settlement of this part of the State ?

I send this to you, Messrs. Editors, as an illustration or verification of an interesting physiological fact. " It is not sufficient that the organizable material be perfect; the influence of certain external agents are requisite to call into action the peculiar properties of the organized structure, viz: heat, light, moisture, electricity," &c.

* It was once the principal staple, but has not been planted for years.

